# Higher Education for Women in America

**Published:** 1891-04-04

**Authors:** 


### Higher Education for Women in America.

"How is it that you American girls are so delightful to talk to—so intelligent, and all that, you know—while your brothers are—well, such bears?" Such was the question asked the other day, by a certain young Englishman, of his fair companion at a dance; and the answer which he received (accompanied, we may hope, by a look which showed that his want of manners had been taken stock of) was this: "Our brothers go into business at fifteen or sixteen, you see; *we* go to college."

We are certainly inclined to believe, as does Max O'Rell, that American girls, as a rule, have a "high old time." And not the least of their advantages is the provision made for them in the way of higher education. Two hundred and seventeen out of the 357 Colleges of Liberal Arts open their doors to women; and besides this, there are 207 colleges and seminaries for women only, scattered over the United States, the grand total of women availing themselves of one or other of these privileges during the year 1888 being 42,663, and the number of degrees given by colleges for women only during the same year, 853. We English people flatter ourselves that we have done something in the way of promoting higher education for women; but this quite takes the shine out of us. "The United States," says the American Commissioner of Education, "has been foremost in this movement; its example has been stimulating to other nations, and the history of what has here been accomplished is the constant subject of inquiry and study on the part of those who are interested in similar efforts in foreign countries." Thankyou, Mr. Commissioner, for the hint. We will put our pride in our pocket, and sit at the feet of this enterprising child of ours.

As was said above, there are 207 colleges or seminaries in America for women only. Seven of these are distinguished from the rest by being modelled upon the usual college system for men, their organization, their standards of instruction, and their admission requirements being practically the same. The largest of these colleges, Wellesley College, Mass., which was opened in 1875, has over 600 students and 77 instructors. Its school of music is located in Music Hall, where there are 38 music rooms, besides a hall for lectures and choral singing. There is also a school of art, the purpose of which is twofold—to provide technical instruction in drawing and painting, and to "supply such acquaintance with the arts in their history, philosophy, and criticism as may profitably supplement the work in other departments of study." The ordinary classical and scientific course in this college takes four years; but students may combine with it the regular study of music, by taking a course of five years instead of four.

The youngest of these colleges is Bryn Mawr, Pennsylvania. It was opened in 1875, with 36 students, and has now more than doubled its numbers. In this college the "group system" is in vogue, every candidate for a degree being required to take two "major courses," which shall be homogeneous, or shall complete each other - such, for instance, as History with Political Science, Mathematics with Physics, or Any Language with Any Language. This is intended to lay the foundation of a specialist's knowledge, while the "minor courses," which must also be taken, supplement the "group," and ensure a liberal training. Bryn Mawr gives five fellowships annually, besides a European fellowship, which entitles its holder to a year's residence at some foreign university, English or Continental. The charge for tuition at these colleges is 100 dollars a-year—say £20; and the average cost of board about 250 dollars; so that a year at college will cost an American girl about £70.

We may mention here the "Harvard Annex," an institution which is at present unique, but which furnishes a precedent which will probably be followed ere long by other universities. The Harvard Annex (opened in 1879) repeats for women the instruction given in Harvard College to men. It has its own laboratories, but no library and no teachers of its own, the college library being open to all students at the Annex, and the entire course of instruction being given by the college teachers. The Annex is more expensive than the colleges we have before mentioned, the annual tuition fees being 200 dollars, and the cost of board and lodging 300 dollars; but it is evidently popular, the number of students steadily increasing year by year. Most of the students are preparing for the work of teaching; but we are told that there is a gradual growth in the proportion of students who are "women of scholarly tastes, with means to gratify them, who come to study under higher auspices simply because they enjoy it." "We have had as yet," says the executive committee, "no flighty students, brought by the novelty of the thing, and very little fragmentary, half-digested work."

We must now take a glance at the remaining 200 colleges or seminaries for women. Many of these have been at work for fifty or sixty years; and we notice that one was opened as early as 1804. A gradual process of alteration and selection has been going on among them. some developing into colleges proper, some gradually confining themselves to the work of preparing students for college, and others making provision for that large class of girls to whom a regular college training is not adapted. More than half of them, however, have the power to confer collegiate degrees; and the course of study in the greater number is of the usual collegiate length, four years. The Commissioner of Education tells us that 64 per cent. belong to some one of the religious denominations, and that this relation has done much to develop that zeal for charitable and missionary labours which characterizes a large proportion of the educated women of the country. We regret that we have not space for fuller details of Lake Erie Female Seminary, Ohio, which, while "laying the foundation for advanced special training according to university methods," gives time for the use of the library, for essay writing, training in household duties, &c. The ordinary daily work of the household (lightened by many appliances for lifting weights and saving steps) is done by the students, one hour daily being set apart for it; and the teachers consider that this plan, which was entered upon at first for the sake of economy, gives healthful exercise, honours labour, and cultivates a taste for home occupations. The seminary is unsectarian, but six missionary societies are supported by the students, who all unite, we are told, in daily worship, and in the study of all movements, religious and philanthropic, for the good of the world.

Passing on to professional schools, we find that there are six medical schools for women in the United States, one of these being homœopathic, and that in 1888 the number of degrees conferred by them was 65. The largest of these schools is the Medical College at Philadelphia, which in 1888 had 160 students. Its president is Dr. Clara Marshall; the annual cost of tuition, board, and lodging, 250 dollars; and the length of the course of study, three years.

We have left ourselves but little space in which to speak of the 217 Colleges of Arts and Science which are open to women as well as men, and can only say here that 16,000 young women avail themselves of the advantages thus offered to them. At the Kansas University, in which special attention is paid to electrical engineering, chemistry, and general physics, a building is to be constructed for the accommodation of lady students; so that before many years have passed, the college will, we presume, turn out fully equipped electrical engineers of the gentler sex. At any rate, nearly 1,000 women attend the Colleges of Agriculture and the Mechanic Arts. In these are taught field, garden, and shop work, surveying, military tactics, industrial art, and household industry. We have no statistics showing which classes are chiefly patronised by girl-students, but will hazard the guess that industrial art and household industry claim the greater number of them, and military tactics, none at all.

